# Systematic analyses of the factors influencing sperm quality in patients with SARS-CoV-2 infection

**DOI:** 10.1038/s41598-024-58797-y

**Published:** 2024-04-07

**Authors:** Guohui Zhang, Weiwei Zhi, Fei Ye, Dongsheng Xiong, Yanan Zhang, Fulin Liu, Yuhong Zhao, Xinrong Du, Yang Wu, Mingxia Hou, Jiu Liu, Jiajing Wei, Yangzhong Silang, Wenming Xu, Jiuzhi Zeng, Shiqi Chen, Weixin Liu

**Affiliations:** 1 Key Laboratory of Reproductive Medicine, Sichuan Provincial Maternity and Child Health Care Hospital, Chengdu, 610045 China; 2 Sichuan Provincial Key Laboratory for Human Disease Gene Study and the Center for Medical Genetics, Sichuan Academy of Medical Sciences and Sichuan Provincial People’s Hospital, University of Electronic Science and Technology, Chengdu, 610072 China; 3 School of Laboratory Medicine, Chengdu Medical College, Chengdu, 610500 China; 4 Chengdu University of Traditional Chinese Medicine, Chengdu, 610075 China; 5 Department of Obstetrics and Gynecology, Joint Laboratory of Reproductive Medicine (SCU-CUHK), Key Laboratory of Obstetric, Gynecologic and Pediatric Diseases and Birth Defects of Ministry of Education, West China Second University Hospital, Sichuan University, Chengdu, 610041 China

**Keywords:** Semen parameters, SARS-CoV-2, Fever, Linear mixed-effects model, Infectious diseases, Reproductive disorders, Risk factors

## Abstract

To figure out how does SARS-CoV-2 affect sperm parameters and what influencing factors affect the recovery of sperm quality after infection? We conducted a prospective cohort study and initially included 122 men with SARS-CoV-2 infection. The longest time to track semen quality after infection is 112 days and 58 eligible patients were included in our study eventually. We subsequently exploited a linear mixed-effects model to statistically analyze their semen parameters at different time points before and after SARS-CoV-2 infection. Semen parameters were significantly reduced after SARS-CoV-2 infection, including total sperm count (211 [147; 347] to 167 [65.0; 258], *P* < 0.001), sperm concentration (69.0 [38.8; 97.0] to 51.0 [25.5; 71.5], *P* < 0.001), total sperm motility (57.5 [52.3; 65.0] to 51.0 [38.5; 56.8], *P* < 0.001), progressive motility (50.0 [46.2; 58.0] to 45.0 [31.5; 52.8], *P* < 0.001). The parameters displayed the greatest diminution within 30 days after SARS-CoV-2 infection, gradually recovered thereafter, and exhibited no significant difference after 90 days compared with prior to COVID-19 infection. In addition, the patients in the group with a low-grade fever showed a declining tendency in semen parameters, but not to a significant degree, whereas those men with a moderate or high fever produced a significant drop in the same parameters. Semen parameters were significantly reduced after SARS-CoV-2 infection, and fever severity during SARS-CoV-2 infection may constitute the main influencing factor in reducing semen parameters in patients after recovery, but the effect is reversible and the semen parameters gradually return to normal with the realization of a new spermatogenic cycle.

## Introduction

In the aftermath of the global pandemic engendered by the novel coronavirus pneumonia (COVID-19) and caused by SARS-CoV-2, medical scientists are now focusing on the mechanisms by which the SARS-CoV-2 virus infects cells through ACE2 and how it generates multi-organ damage in humans. SARS-CoV-2 enters cells through binding and membrane fusion with ACE2 on the cell membrane, mediated by the spike (S) protein receptor-binding domain, and the trans-membrane serine protease 2 (TMPRSS2) and furin protease are also involved in this process^1^. Due to the abundant expression of ACE2 in the lung, the virus is most likely to invade lung tissue, causing acute respiratory distress syndrome (ARDS) in severe cases. Uncontrolled inflammatory immune responses, high levels of cytokines, and multi-organ failure can then occur and produce high mortality rates. Thus, other organs such as liver, intestines, brain, eyes, heart, blood vessels, and testes can be severely damaged by SARS-CoV-2^2^. ACE2 is also highly expressed in testicular tissue^3,4^, and the proportion of ACE2-positive cells in the testes is even significantly higher than that in the lungs. TMPRSS2 expression is also localized in the male reproductive system, indicating the testes as potential organs at high risk of SARS-CoV-2 infection^5^. Single-cell sequencing data from human testes revealed wide expression of ACE2 in Sertoli cells, Leydig cells, and germ cells at different developmental stages^6^, while in sperm, ACE2 was primarily detected in the flagellar mid-piece and the post-acrosomal region^7^. These results indicate that ACE2 may serve as a receptor mediating SARS-CoV-2 invasion into testicular cells and that this process causes injury to the testes and negatively affects spermatogenesis.

Previous investigators demonstrated that SARS-CoV-1 infection generated problematic complications within the reproductive system^8,9^, and that SARS-CoV-2 invaded human cell populations through the same receptor (ACE2) that was extensively expressed in multiple organs of the human body. In one study, two SARS-CoV-2-positive testicular samples were detected in five samples taken from COVID-19 patients^10^, and in another, the authors detected three SARS-CoV-2-positive testicular samples in 26 patients^11^, suggesting an ability of SARS-CoV-2 to invade and damage the testes. Research data indicated that viral orchitis is caused by SARS-CoV-2 and that it is manifested as a large number of degenerative germ cells in the seminiferous tubules, swelling and vacuolation in Sertoli cells, and infiltration of a large number of inflammatory cells (including T lymphocytes, B lymphocytes, and macrophages) in the testicular interstitium and seminiferous tubules; more severe cases even displayed features of Sertoli cell-only syndrome^10,12^. Researchers also uncovered pathological manifestations of autoimmune orchitis in COVID-19 patients^13^, with most of these signs accompanied by epididymitis. However, controversies remain as to whether testicular damage is caused by the direct actions of the virus or by virally induced autoimmune orchitis, and the specific molecular mechanisms underlying testicular disruption remain unclear.

Although injury to the testes due to SARS-CoV-2 has been observed, it is debatable whether the virus can be determined in semen. In one study of 38 semen samples, six cases of SARS-CoV-2-positivity were identified, two of which were demonstrated in semen from patients who recovered from COVID-19^14^. However, other studies revealed that viral mRNA was absent in semen^10,15–17^. Although changes in semen parameters (including reduced semen volume, sperm concentration, and sperm count) were determined in men with testicular damage and SARS-CoV-2 infection^13,15^, the impacts on sperm motility, viability, and morphology are still disputed. It is therefore currently unclear whether the decrease in semen parameters in infected individuals is due to the viral infection itself or the febrile symptoms. The results of meta-analysis additionally revealed a large heterogeneity in the literature, which may have affected the evaluation of the effects of SARS-CoV-2 on sperm motility^18^. Normal spermatogenesis requires approximately 3 months, and semen volume, concentration, and sperm motility fluctuate under physiological conditions during this timeframe. Therefore, larger sample sizes and longer time-scales are needed to analyze the effects of SARS-CoV-2 infection on spermatogenesis and sperm quality. In addition, SARS-CoV-2 activates cellular oxidative stress, causing sperm DNA fragmentation that is associated with poor embryonic development, reduced implantation rates, and higher miscarriage rates^19–21^. The observation of pregnancy outcomes is therefore also clinically relevant. However, there is a paucity of studies on the long-term effects, potential for sustained sperm quality, and pregnancy outcomes in recovered COVID-19 patients. Our study comprised 58 patients with SARS-CoV-2 infection at our hospital, and we statistically analyzed their clinical characteristics and semen parameters before and after SARS-CoV-2 infection. We collected semen samples several times from these men and analyzed them after the men showed infection with SARS-CoV-2; our latest detection time was 112 days after infection. We expected to clarify the actions of SARS-CoV-2 infection on semen parameters through the present study and further explored the recovery time and potential influencing factors on male sperm quality after SARS-CoV-2 infection (with the latter including fever), thus providing useful guidance in the clinical treatment and assisted reproductive outcomes of patients with SARS-CoV-2 infection.

## Results

### Current studies on the effects of SARS-CoV-2 infection on semen parameters

**Figure 1:**
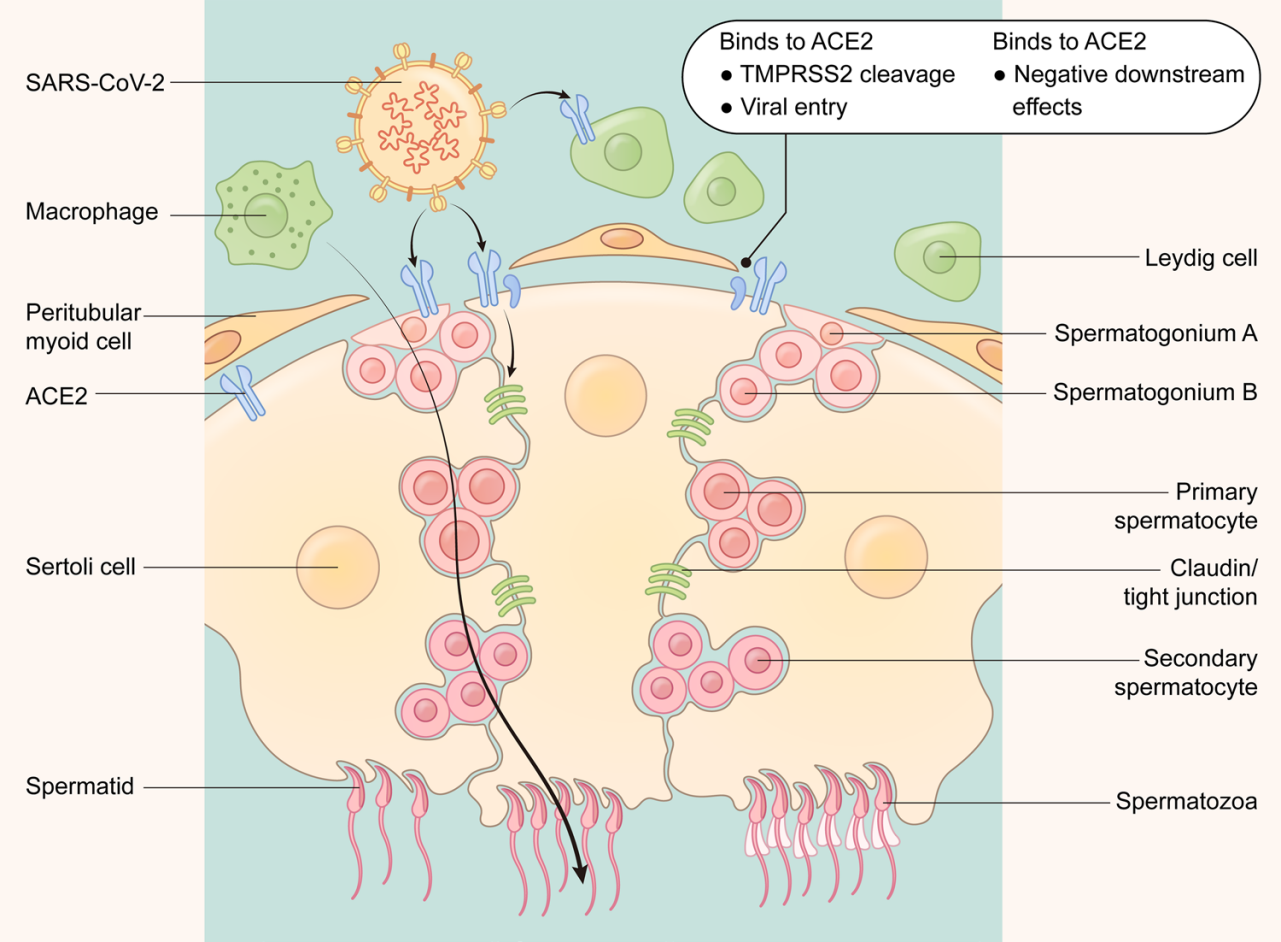
SARS-CoV-2 invades and damages the testes through the widely expressed receptors ACE2 on testicular cells. Various cell types, including leydig cells, sertoli cells, and germ cells with different developing stages widely express ACE2, which medicates the intrusion of SARS-CoV-2 through the blood–testis barrier together with TMPRSS2.

**Figure 2:**
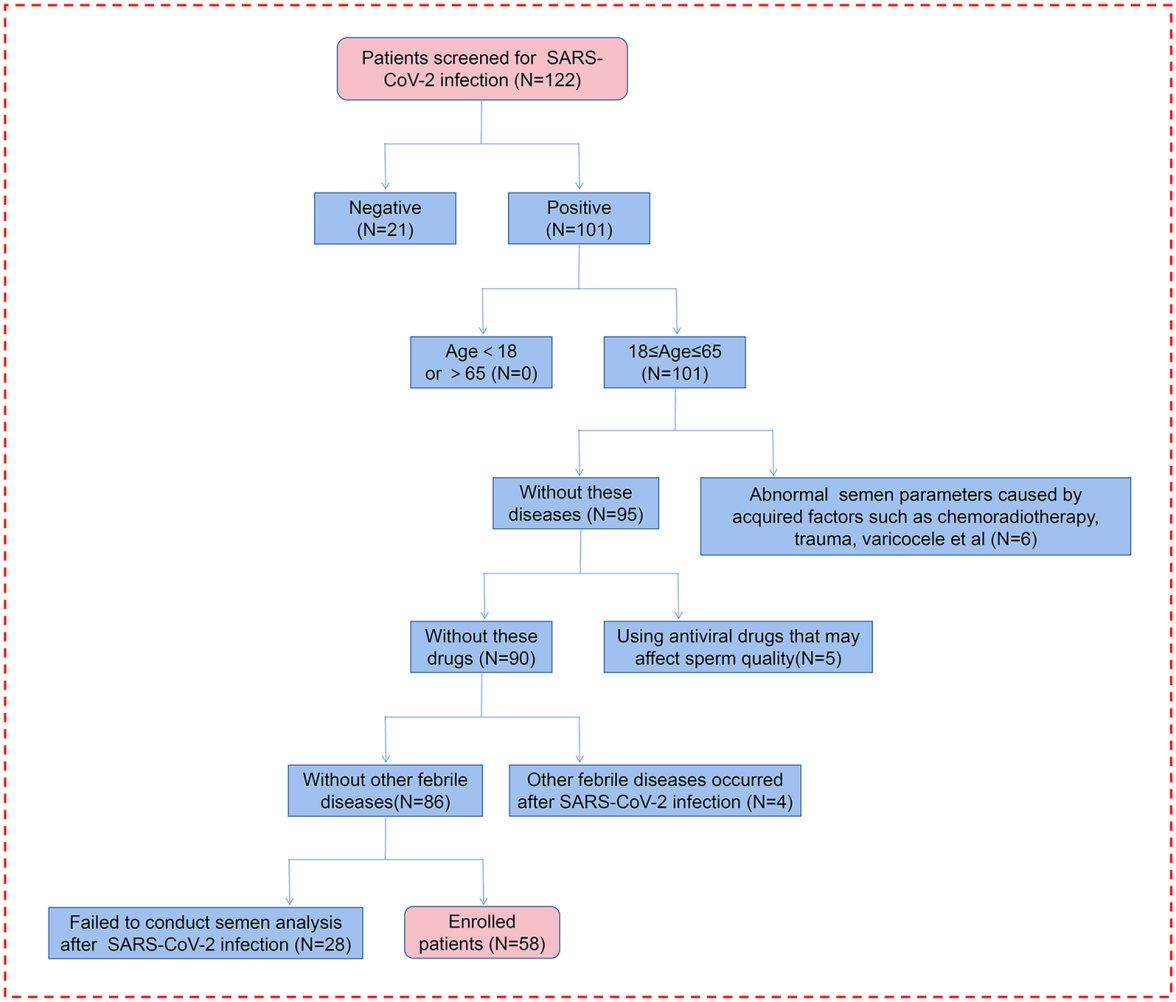
The flow for screening the patients involved in our study.

Previous studies have indicated that SARS-CoV-2 invades body cells through membrane binding and fusion mediated by ACE2 and TMPRSS2^1^, subsequently causing orchitis and injury^10,12^ (Fig. 1). To clarify the actions of SARS-CoV-2 infection on sperm parameters and recovery after disease, we summarized recent studies on the impact of SARS-CoV-2 infection on sperm quality and found that these investigators reported relatively similar conclusions regarding the effects of SARS-CoV-2 infection on semen parameters^15,22–29^ (Table 1). Most studies revealed that semen parameters that included total sperm count, sperm concentration, and total motility were significantly reduced within 3 months after recovery, although no changes were found with respect to semen volume. There is, however, controversy regarding changes in progressive motility, as some studies indicate a decrease, while others suggest no significant changes after recovery. For example, no impact was observed on sperm survival or testosterone concentrations, except in one study that showed a decrease in blood testosterone after infection^29^. Furthermore, none of these studies indicated the presence of SARS-CoV-2 in semen, suggesting that the reduction in semen parameters caused by SARS-CoV-2 infection may not be due to a direct action of the virus on sperm. Considering the heterogeneity of these studies (such as most of the studies were non-self-controlled, and that there has been no long-term or systematic tracking of the recovery time of males after SARS-CoV-2 infection), additional systematic studies need to be conducted to evaluate these scientific questions. Therefore, our study aim was to improve upon these shortcomings. We included 58 patients with SARS-CoV-2 infection in our analysis of semen parameters and collected multiple semen samples before and after infection (the detailed screening process for our study patients is displayed in Fig. 2). Samples were collected at various time points, i.e., at 1, 2, and 3 months and longer after SARS-CoV-2 infection, with the longest collection time at 109 days after recovery. We aimed to clarify the effects of SARS-CoV-2 infection on sperm parameters and on the recovery of semen quality, providing guidance for clinical treatment and assisted reproduction practices of the patients who exhibited altered sperm quality.

**Table 1:** Study conclusions of the recovery of sperm parameters after rehabilitation of SARS-CoV-2 infection.

Study type	Number of participants		Self control	Average age		Sampling time (after recovery)		Semen parameters							Testosterone	Virus in semen?	Conclusion (Does it affect semen parameters?)
	Control	Patients		Control	Patients	Fir	Sec	Semen volume	Sperm counts	Sperm concentrations	Sperm motility	Progressive motility	Vitality	Abnormal sperm morphology			
Prospective study^22^	50	41	No	26.5	26.0	56 days	84 days	NS	Reduced	Reduced	Reduced	Reduced	NS	NS	NS	NA	YES
Prospective study^23^	21	21	Yes	32		51 days		Reduced	NS	NS	Reduced	Reduced	NA	Elevated	Reduced	NA	YES
Prospective study^24^	45	36	No	31.49	31.75	83 days	175 days	NS	Reduced	NS	NS	NS	NA	NA	NA	NA	YES
Prospective study^25^	36	22	No	29	29	91.5 days		Reduced	NA	Reduced	NS	NS	NS	NS	NS	NA	YES
Cohort study^15^	14	4	No	33.4	40.8	25.5 days		NS	Reduced	Reduced	Reduced	Reduced	NA	NA	NA	NO	YES
Prospective study^26^	145	55	No	30.69	31.15	77.3 days		NS	Reduced	Reduced	Reduced	NS	NA	NA	NA	NO	YES
Prospective study^27^	24	24	Yes	34.7		111.5 days		NS	NA	NS	Reduced	NS	NA	NA	NA	NA	YES
Retrospective study^28^	26	26	Yes	33		More than 4 months		NS	NS	NS	NS	NS	NA	Elevated	NA	NA	YES
Cross sectionalstudy^29^	29	29	Yes	31.21		4.52 months		NS	NS	NS	NS	NS	NA	NA	NS	NA	NO

*NS* no significance, *NA* not available.

### Analysis of basic clinical characteristics of the study patients

We conducted a comprehensive analysis of the basic clinical information of the 58 patients involved in this study, including age, BMI, timing of semen analysis before and after SARS-CoV-2 infection, and duration of infection. Additionally, following the criteria outlined in the 10th edition of the COVID-19 Diagnosis and Treatment Guidelines, we categorized the patients into mild and moderate cases, with no occurrences of severe or critical cases in our study. We meticulously recorded the temperature and duration of fever, classifying patients into low, moderate, and high fever groups. This classification was undertaken to investigate the impact of fever on sperm parameters and the post-infection recovery, as this factor remains a subject of considerable debate. Further details of the clinical features were presented in Table 2.

**Table 2:** Clinical features of the patients involved in this study.

Variable	Value (n = 58)
Age^1^	31.6 (3.24)
BMI^1^	23.8 (3.24)
Days of semen analysis before SARS-CoV-2 infection^2^	74.5 [24.5, 238]
Days of semen analysis after SARS-CoV-2 infection^2^	50.0 [32.8, 75.8]
≤ 30 days^3^	13 (22.4%)
31–60 days^3^	28 (48.3%)
> 60 days^3^	17 (29.3%)
Duration of infection^2^	4.00 [3.00, 6.00]
Maximum fever temperature^2^	38.5 [38.0, 38.8]
Fever days^2^	2.50 [2.00, 3.00]
Fever levels	
Low fever^3^	19 (32.8%)
Moderate fever^3^	6 (10.3%)
High fever^3^	33 (56.9%)
Clinical classification	
Mild^3^	53 (91.4%)
Moderate^3^	5 (8.62%)
Self-feeling	
Mild^3^	25 (43.1%)
Moderate^3^	29 (50.0%)
Severe^3^	4 (6.90%)
Muscle soreness^3^	48 (82.8%)
Dry cough^3^	36 (62.1%)
Sore throat^3^	44 (75.9%)
Parageusia^3^	24 (41.4%)
Heterosmia^3^	13 (22.4%)
Diarrhea^3^	9 (15.5%)
Testicular discomfort^3^	0 (0%)
Dizziness and headache^3^	28 (48.3%)
Expectoration^3^	19 (32.8%)
Nasal obstruction^3^	21 (36.2%)
Short of breath^3^	14 (24.1%)
Nausea and vomiting^3^	6 (10.3%)
Eye discomfort^3^	1 (1.72%)
Hoarse voice^3^	1 (1.72%)
Palpitate^3^	5 (8.62%)
Lumbodynia^3^	11 (19.0%)
Feeble^3^	26 (44.8%)
Chest tightness^3^	6 (10.3%)

^1^Mean (standard deviation).

^2^Median [Quartile 1, Quartile 3].

^3^Number [percentage].

### Changes in semen parameters of males with SARS-CoV-2 infection

Previous studies have depicted a SARS-CoV-2 infection as causing inflammation to the testes, but the specific effects of SARS-CoV-2 infection on semen parameters are still debated. We conducted a comparative analysis of indices in semen from 58 male patients before and after SARS-CoV-2 infection (as shown in Table 3) and noted no significant differences in the semen volume between the two groups, while the median total sperm count and concentration was reduced after infection. The proportions reflecting total sperm motility and progressive motility were significantly reduced after infection, while the proportion of immotile sperm was increased. The sperm survival rate and the normal morphology rate were also reduced, mainly manifested as increased head defect, however, showed no differences in the numbers of sperm with neck, mid-piece, or tail defects. In addition, we observed no differences in the number of round cells, anti-sperm antibodies, semen liquefaction time, or viscosity before vs. after SARS-CoV-2 infection. More detailed information is presented in Table 3.

**Table 3:** Semen parameters of the patients before and after SARS-CoV-2 infection.

Semen parameters	Before infection	After infection	*P* value
Total count^1,2^	211 [147; 347]	167 [65.0; 258]	< 0.001**
Concentration (× 10^6^/ml)^1,2^	69.0 [38.8; 97.0]	51.0 [25.5; 71.5]	< 0.001**
Semen volume (ml)^1^	3.55 [2.42; 4.60]	3.65 [2.62; 4.77]	0.88
PH degree^1,2^	7.20 [7.20; 7.40]	7.20 [7.20; 7.60]	0.042*
Abstinence days^1^	4.00 [3.00; 5.00]	3.00 [3.00; 4.00]	0.138
Normal morphology (%)^1,2^	2.50 [1.00; 4.00]	2.00 [1.00; 2.79]	0.017**
Head defect (%)^1,2^	97.5 [96.0; 99.0]	98.0 [97.0; 99.0]	0.019**
Neck or middle piece defect (%)^1^	33.0 [28.8; 36.0]	33.0 [31.0; 35.0]	0.559
Tail defect (%)^1^	11.0 [10.0; 13.0]	11.0 [10.0; 12.0]	0.79
Teratozoospermia index^1^	1.46 [1.40; 1.49]	1.45 [1.43; 1.48]	0.767
Sperm deformity index^1^	1.43 [1.36; 1.46]	1.43 [1.40; 1.45]	0.436
Sperm coating antibody^1^	4.00 [2.75; 5.00]	4.00 [3.00; 5.00]	0.493
Total motility (%)^1,2^	57.5 [52.3; 65.0]	51.0 [38.5; 56.8]	< 0.001**
Progressive motility (%)^1,2^	50.0 [46.2; 58.0]	45.0 [31.5; 52.8]	< 0.001**
Non-progressive motility (%)^1^	6.00 [4.00; 7.00]	5.00 [4.00; 7.00]	0.826
Immobile (%)^1,2^	42.0 [35.0; 46.6]	49.0 [43.2; 61.5]	< 0.001**
Survival rate (%)^1,2^	85.0 [85.0; 88.0]	85.0 [82.0; 85.2]	0.008**
Round cell (> 1)^3^	7 (12.1%)	9 (15.5%)	0.752
Liquefaction time (< 30)^3^	5 (8.62%)	2 (3.45%)	0.449
Viscosity (> 2 cm)^3^	6 (10.3%)	3 (5.17%)	0.505

^1^Median [Quartile 1, Quartile 3].

^2^*P < 0.05, **P < 0.01.

^3^Number (percentage).

### Trends in semen quality before vs. after SARS-CoV-2 infection (using a linear mixed-effects model)

**Figure 3:**
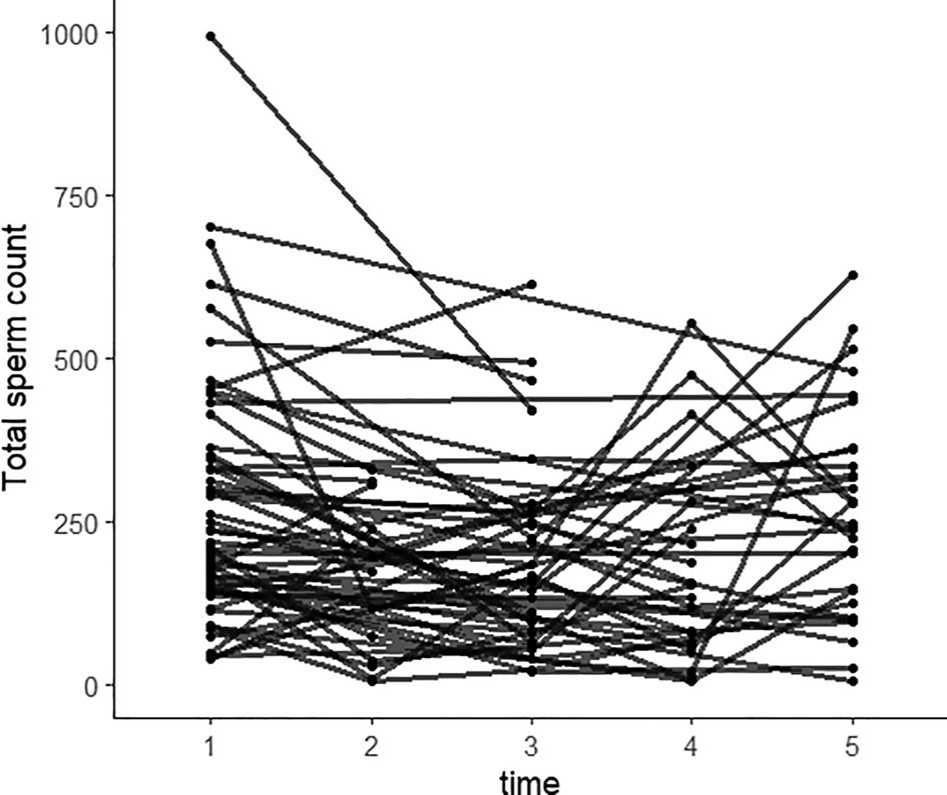
Observations of total sperm count at different time points of the patients. 1 represents pre-SARS-CoV-2 infection; 2 represents the observation time was within 30 days after infection; 3 represents the observation time was between 31 and 60 days after infection; 4 represents the observation time was between 61 and 90 days after infection; 5 represents the observation time was more than 91 days.

**Figure 4:**
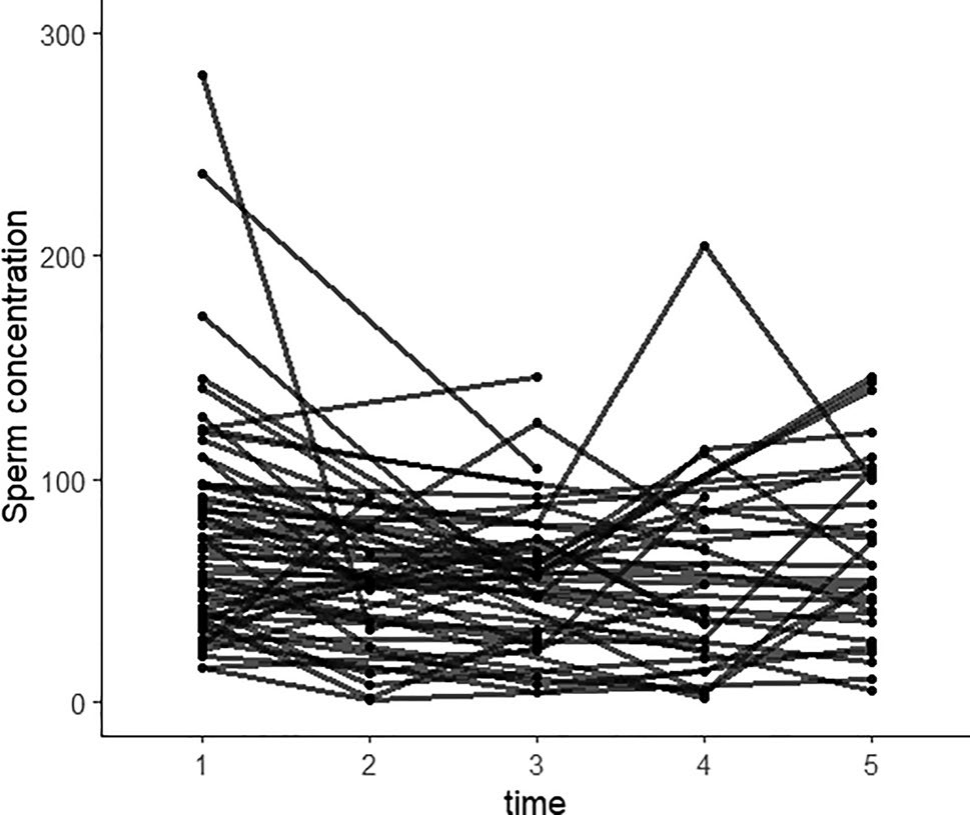
Observations of sperm concentration at different time points of the patients. 1 represents pre-SARS-CoV-2 infection; 2 represents the observation time was within 30 days after infection; 3 represents the observation time was between 31 and 60 days after infection; 4 represents the observation time was between 61 and 90 days after infection; 5 represents the observation time was more than 91 days.

**Figure 5:**
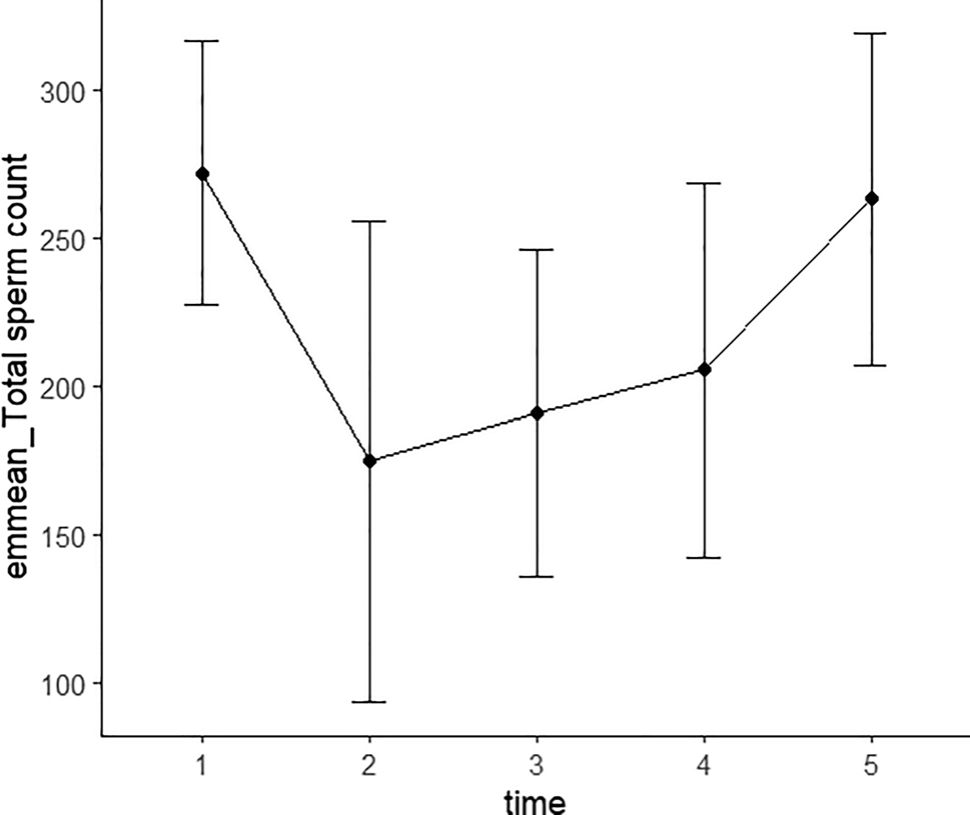
The estimated marginal means (EMMs) of all the observations of total sperm count at different time points of the patients. 1 represents pre-SARS-CoV-2 infection; 2 represents the observation time was within 30 days after infection; 3 represents the observation time was between 31 and 60 days after infection; 4 represents the observation time was between 61 and 90 days after infection; 5 represents the observation time was more than 91 days.

**Figure 6:**
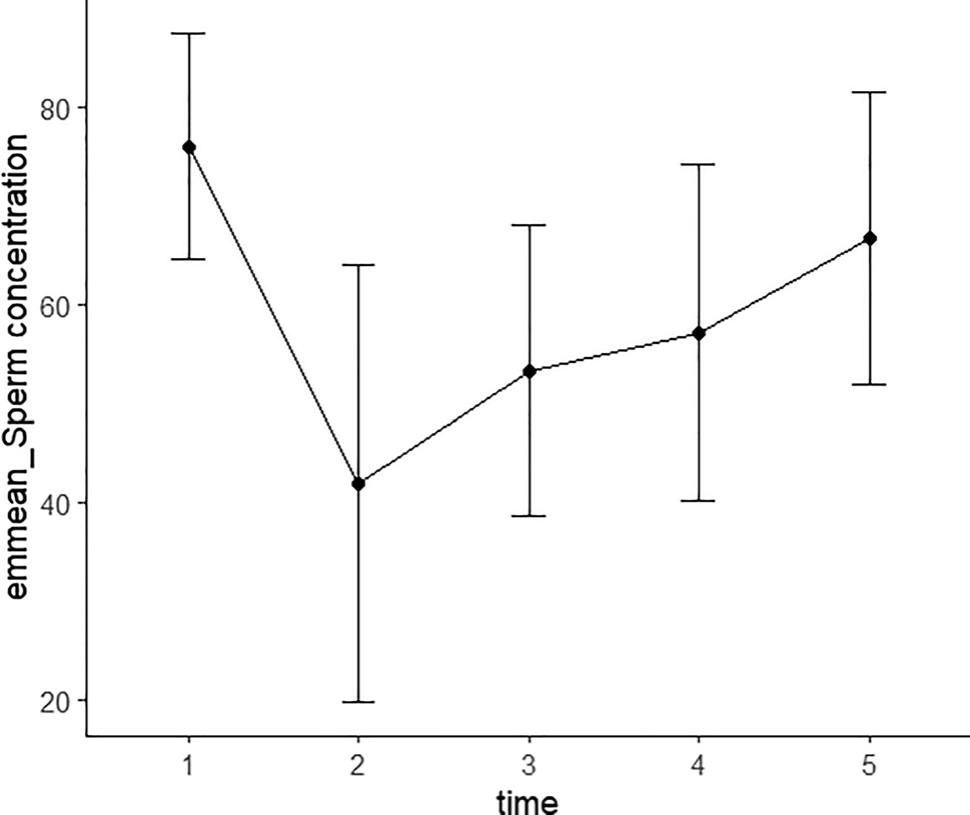
The estimated marginal means (EMMs) of all the observations of sperm concentration at different time points of the patients. 1 represents pre-SARS-CoV-2 infection; 2 represents the observation time was within 30 days after infection; 3 represents the observation time was between 31 and 60 days after infection; 4 represents the observation time was between 61 and 90 days after infection; 5 represents the observation time was more than 91 days.

To further analyze the changes in and restoration of semen quality after SARS-CoV-2 infection, we constructed a linear mixed-effects model for the semen parameters that were significantly decreased after SARS-CoV-2 infection. Parameters included total sperm count, sperm concentration, percentage of normal sperm, proportions of sperm with head defects and those showing normal motility and progressive motility, the proportion of immobile sperm, and sperm survival rate. Time was designated as the fixed effect and participant ID as the random effect. We divided observations into different groups according to detection time: 1 represented pre-SARS-CoV-2 infection; 2, the observation time within 30 days after infection; 3, the observation time between 30 and 60 days after infection; 4, the observation time between 60 and 90 days after infection, and 5 represented the observation time of over 90 days. The numbers of observations at 1–5 time points were 58, 13, 32, 23, and 31, respectively, and Figs. 3 and 4 display the observations of total sperm counts and sperm concentrations at different time points. The changing trends in semen quality before vs. after infection are shown in Table 4. Figures 5 and 6 show the EMMs (estimated marginal means) with corresponding standard errors (SE) across time for sperm count and concentration. It is notable that the greatest diminution in sperm count and concentration occurred within 30 days after SARS-CoV-2 infection, followed by a gradual recovery after 30 days, and we noted no difference between before and after SARS-CoV-2 infection 90 days later. Intriguingly, the proportion of normal sperm fell and the proportion of sperm with head defects rose significantly between 30 and 60 days after infection and also showed a tendency to recover after 60 days (Table 4, Figs. S1 and S2). Moreover, assessments of sperm motility indicated that the greatest decrease in total motility occurred within 30 days after infection, followed by a gradual recovery and that motility returned to normal after 60 days (Table 4, Fig. S3). There was, however, a slight decrease in sperm motility after 90 days, which may have been attributable to the elevation in the proportion of immobile sperm after 90 days (Table 4, Fig. S4). The proportion of sperm exhibiting progressive motility significantly decreased within 30 days after infection and subsequently displayed a recovery between 30 and 60 days—with no significant difference between the two groups before vs. after 60 days of infection (Table 4, Fig. S5). Sperm survival rate decreased most significantly within 30 days and recovered after 30 days (Table 4, Fig. S6). Figures S7–S12 depict the distributions of observed values with respect to the proportions of normal sperm, sperm with head defects, motile sperm, progressively motile sperm, and immobile sperm, and sperm survival rate at different time points. These results indicated that the most significant diminutions in various semen parameters were observed within 30 days after infection, followed by a gradual recovery. Despite the variation in the recoveries of different indicators, all parameters basically returned to normal after 90 days.

**Table 4:** The changes in trend of semen quality over time before and after SARS-CoV-2 infection (linear mixed effect model).

	Sperm count	Sperm concentration	Normal morphology	Head defect	Total motility	Progressive motility	Immobile	Survival rate
Constant^1^	272.14 (22.20)	76.08 (5.73)	2.57 (0.22)	96.84 (0.33)	58.90 (1.87)	52.23 (1.93)	40.79 (1.86)	84.86 (1.07)
TP1-TP2^1,2,^	− 97.26 (40.81)*	− 34.09 (11.38)**	0.19 (0.50)	− 0.15 (0.77)	− 16.74 (4.00)***	− 15.10 (3.97)***	16.98 (4.01)***	− 8.26 (2.30)***
TP1-TP3^1,2^	− 81.09 (27.99)**	− 22.64 (7.84)**	− 0.85 (0.34)*	1.37 (0.53)**	− 9.13 (2.78)**	− 8.24 (2.75)**	9.37 (2.80)**	− 2.05 (1.49)
TP1-TP4^1,2^	− 66.62 (31.99)*	− 18.80 (8.91)*	− 0.44 (0.38)	1.14 (0.59)	− 3.43 (3.15)	− 5.04 (3.12)	3.71 (3.17)	− 1.56 (1.71)
TP1-TP5^1,2^	− 8.94 (28.43)	− 9.25 (7.93)	− 0.13 (0.34)	0.70 (0.53)	− 6.59 (2.81)*	− 5.27 (2.78)	6.98 (2.83)*	− 0.93 (1.52)
Observations	156	157	155	154	157	157	157	149
Log likelihood	− 1002.77	− 803.16	− 296.11	− 356.14	− 632.59	− 634.87	− 632.61	− 507.97
Akaike Inf. Crit	2019.54	1620.33	606.21	726.29	1279.18	1283.74	1279.22	1029.93
Bayesian Inf. Crit	2040.89	1641.72	627.52	747.54	1300.57	1305.13	1300.61	1050.96

^1^Values indicate the estimated effect (β) and corresponding standard error (SE).

^2^TP1 (time point 1) represents the time point of pre-SARS-CoV-2 infection; TP2 represents the observation time was within 30 days after infection; TP3 represents the observation time was between 31 and 60 days after infection; TP4 represents the observation time was between 61 and 90 days after infection; TP5 represents the observation time was more than 91 days.

*P < 0.05, **P < 0.01, ***P < 0.001.

Interestingly, compared to the control group, the malondialdehyde (MDA) concentration in patients' sperm significantly increased within 30 days post infection, but showed no significant difference after 90 days (Fig. S13C). However, the expression of COX-IV (the mitochondrial respiratory chain key enzyme) showed no significant difference from the control group either within 30 days or 90 days post infection (Fig. S13A,B). Moreover, immunofluorescence results indicated that the distribution of COX-IV in SARS-CoV-2 infected patients' sperm was more diffuse compared to the control group (Fig. S13A), unlike the well-localization in mitochondrial sheath in the control group, suggesting potential abnormalities in mitochondrial morphology in SARS-CoV-2 infected patients.

### High core body temperature during SARS-CoV-2 infection contributes to the changes in semen parameters after recovery

To analyze the factors that influenced semen parameters after recovery from SARS-CoV-2 infection, we allotted the patients to different subgroups based on their clinical symptoms and subsequently analyzed their semen parameters before and after infection. When we allotted 58 patients to the two groups of those with low-grade fevers (19) and those with moderate-to-high fevers (39) according to fever severity, we uncovered no significant differences in age, BMI, timing of semen detection before vs. after infection, duration of disease, or other clinical symptoms except for fever (Table S1). Results from the 19 patients in the low-grade fever group showed no significant differences in semen parameters other than anti-sperm antibody levels before vs. after infection (Table 5). However, analysis of the semen parameters after infection in the 39 patients with moderate-to-high fever showed a significant reduction in total sperm count, sperm concentration, normal sperm proportion, total motility, progressive motility, and sperm survival rate. We also noted an elevation in the proportions of sperm with head defects and immotile sperm (Table 6). These results suggested that moderate-to-high fever may constitute a risk factor for the decline in semen parameters after recovery from SARS-CoV-2 infection, while low-grade fevers exerted a much smaller impact.

**Table 5:** Comparative analysis of semen parameters in patients with low fever before and after SARS-CoV-2 infection.

Semen parameters	Before infection	After infection	*P* value
Total count^1^	191 [76.2, 323]	184 [65.1, 278]	0.679
Concentration (× 10^6^/ml)^1^	73.0 [33.0, 104]	58.0 [25.0, 76.5]	0.184
Semen volume (ml)^2^	3.07 (1.26)	3.54 (1.30)	0.077
PH degree^1^	7.20 [7.20, 7.40]	7.20 [7.20, 7.60]	0.798
Abstinence days^1^	4.00 [3.00, 5.00]	3.00 [3.00, 4.00]	0.083
Normal morphology (%)^1^	2.50 [1.00, 3.35]	2.00 [1.00, 3.38]	0.608
Head defect (%)^1^	97.8 [95.2, 99.0]	98.0 [96.1, 99.0]	0.649
Neck or middle piece defect (%)^1^	34.0 [16.7, 36.0]	33.0 [31.2, 37.0]	0.459
Tail defect (%)^1^	10.0 [10.0, 12.7]	10.5 [10.0, 11.8]	0.753
Teratozoospermia index^1^	1.46 [1.25, 1.48]	1.46 [1.43, 1.49]	0.492
Sperm deformity index^1^	1.44 [1.23, 1.44]	1.42 [1.40, 1.45]	0.394
Sperm coating antibody^1,3^	3.00 [2.50, 5.00]	5.00 [4.00, 5.00]	0.028*
Total motility (%)^1^	55.0 [51.5, 63.5]	53.0 [49.5, 56.0]	0.344
Progressive motility (%)^2^	49.1 (9.32)	47.0 (18.5)	0.52
Non-progressive motility (%)^1^	6.00 [5.00, 7.45]	5.00 [4.00, 5.50]	0.063
Immobile (%)^1^	45.0 [36.5, 48.5]	47.0 [44.0, 50.5]	0.344
Survival rate (%)^1^	85.0 [84.0, 87.0]	86.0 [85.0, 88.0]	0.343
Round cell (> 1)^4^	2 (10.5%)	4 (21.1%)	0.617
Liquefaction time (= 30)^4^	2 (10.5%)	1 (5.26%)	> 0.999
Viscosity (> 2)^4^	1 (5.26%)	0 (0.00%)	> 0.999

^1^Median [Quartile 1, Quartile 3].

^2^Mean (standard deviation).

^3^*P < 0.05.

^4^Number (percentage).

**Table 6:** Comparative analysis of semen parameters in patients with moderate to high fever before and after SARS-CoV-2 infection.

Semen parameters	Before infection	Before infection	*P* value
Total count^1,2^	238 [151, 350]	145 [65.3, 254]	< 0.001***
Concentration (× 10^6^/ml)^1,2^	68.0 [42.5, 91.0]	47.0 [27.0, 65.0]	< 0.001***
Semen volume (ml)^1^	4.20 [2.85, 4.85]	3.80 [2.95, 4.80]	0.23
PH degree^1,2^	7.20 [7.20, 7.25]	7.20 [7.20, 7.65]	0.036*
Abstinence days^1^	4.00 [3.00, 5.00]	3.00 [3.00, 4.00]	0.371
Normal morphology (%)^1,2^	2.50 [1.25, 4.00]	2.00 [1.00, 2.00]	0.013*
Head defect (%)^1,2^	97.2 [96.0, 98.8]	98.0 [98.0, 99.0]	0.007**
Neck or middle piece defect (%)^1^	33.0 [29.0, 36.0]	33.0 [30.5, 35.0]	0.91
Tail defect (%)^1^	11.0 [10.0, 13.0]	11.0 [10.0, 12.0]	0.643
Teratozoospermia index^1^	1.46 [1.40, 1.49]	1.45 [1.42, 1.48]	0.875
Sperm deformity index^1^	1.42 [1.36, 1.46]	1.43 [1.40, 1.46]	0.688
Sperm coating antibody^1^	4.00 [3.00, 5.00]	3.00 [3.00, 5.00]	0.688
Total motility (%)^1,2^	58.0 [54.0, 67.5]	49.0 [35.0, 58.0]	< 0.001***
Progressive motility (%)^1,2,3^	53.7 (13.1)	40.8 (17.5)	< 0.001***
Non-progressive motility (%)^1^	5.00 [3.00, 6.00]	6.00 [5.00, 7.00]	0.315
Immobile (%)^1,2^	41.0 [32.5, 46.0]	51.0 [42.0, 65.0]	< 0.001***
Survival rate (%)^1,2^	86.0 [85.0, 88.2]	84.0 [79.0, 85.0]	< 0.001***
Round cell (> 1)^4^	5 (12.8%)	5 (12.8%)	> 0.999
Liquefaction time (= 30)^4^	3 (7.69%)	1 (2.56%)	0.617
Viscosity (> 2)	5 (12.8%)	3 (7.69%)	0.724

^1^Median [Quartile 1, Quartile 3].

^2^*P < 0.05, **P < 0.01, ***P < 0.001.

^3^Mean (standard deviation).

^4^Number (percentage).

## Discussion

### How does viral infection affect sperm quality?

Spermatogenesis is a major physiological event that occurs in the seminiferous epithelium, and it encompasses four phases^30^: spermatogonial stem cells first self-renew and spermatogonia proliferate and differentiate into primary spermatocytes^31^; primary spermatocytes undergo meiosis to form round spermatids^32^; round spermatids transform to elongated spermatids and spermatozoa^33^; and finally spermatozoa are released into the seminiferous tubule lumen. This is the normal process that only takes place in the testis. However, congenital disorders of sexual development, hormonal imbalances, anatomical anomalies of the reproductive system, acquired traumas, drug-induced injury, radiation exposure, bacterial and viral infections, and genetic factors may affect the orderly process of sperm production in the seminiferous tubules, leading to male infertility^34^. More than 27 viruses are found in human semen^35^, and several viruses exert a negative effect on male reproduction and spermatogenesis^36^. Jorge Hallak^37^ and colleagues' review extensively elucidates the evidence of the presence of 12 viruses in the male reproductive tract and their adverse effects. The review clarifies the routes of infection, target organs and cells, the prevalence, and patterns of viral shedding in semen, as well as diagnostic/testing and treatment strategies. Acute bacterial infections generally affect the epididymis and accessory glands via the ascending urogenital tract, while viral infection predominantly perturbs the testes through blood circulation^38^. Mumps virus, human immunodeficiency virus (HIV), Zika virus, and coronaviruses can all cause orchitis. Meanwhile, hepatitis B virus (HBV), hepatitis C virus (HCV), herpes simplex virus (HSV), HIV, influenza virus, and Zika virus can lead to changes in sperm parameters. The most of the viruses primarily target the testes, with only a few, such as HIV, human papillomavirus (HPV), HSV, and Zika virus, directly affecting accessory gland organs like the epididymis, vas deferens, seminal vesicles, prostate, and penis. Detection of viruses in tissues suggests their potential to directly damage the male reproductive system and semen parameters. Acute viral infections have also been confirmed to induce systemic reactions, thereby impacting sperm quality systematically^37^. For example, there is evidence to indicate that influenza damages the integrity of sperm DNA^39–41^ and that impaired sperm quality can be detected from four to 11 weeks after fever. It is speculated that the potential mechanisms underlying the untoward effects on sperm may entail (I) fever that causes increased testicular temperature and damages germ cell lines, and (II) viruses that induce orchitis and impair the exocrine and endocrine functions of the testes. Therefore, the mechanisms by which different viral infections impair semen parameters and male reproductive function are highly heterogeneous and not well-defined. A better understanding of the infection pathways and target cells in the male reproductive tract is crucial for devising appropriate treatment and prevention strategies. Additionally, existing data indicate that Ebola virus, HBV, HCV, HSV, HIV, HPV, and Zika virus can be detected in semen, while there is no clear evidence for the presence of influenza virus, mumps virus, and coronaviruses in the semen of infected individuals. Research on this aspect of the novel coronavirus is not sufficiently deep and is controversial. Most studies have not provided data on the presence of SARS-CoV-2 in semen, with a few reporting no detection of the virus in the semen of SARS-CoV-2-infected patients. Although these viruses detectable in semen can be transmitted sexually (except for Zika virus), their presence does not necessarily indicate infectivity, as this requires a certain viral load and titer. Further in-depth research is needed to clarify these aspects and provide a better understanding of the etiology, infection pathways, and target tissues.

### SARS-CoV-2 invades testes through ACE2 receptors and causes orchitis

The widespread expression of the receptor protein ACE2 that mediates the entrance of SARS-CoV-2 into Sertoli cells, Leydig cells, and germ cells at different developmental stages^6^, facilitates viral invasion and allows testicular tissue function to be compromised. The study conducted by Jorge Hallak et al.^42^ provides robust support for this conclusion. They observed the expression of ACE2 and TMPRSS2 in all cases, unaffected by age. These immunofluorescence staining were concurrently present in Leydig cells, Sertoli cells, spermatogonia, endothelial cells, and fibroblasts. Even in atrophic tubules, ACE2 and TMPRSS2 were co-expressed. SARS-CoV-2 enters cells through binding to ACE2 on the cell surface, mediated by the receptor-binding domain of the spike protein, and membrane fusion, with the cell transmembrane serine protease 2 (TMPRSS2) participating in this process^1^. This establishes the molecular basis for the invasion and damage of testicular tissue by SARS-CoV-2. The findings by Jorge Hallak et al.^42^ further confirm this, as electron microscopy results reveal the presence of viral particles in various cells, including supporting cells, interstitial cells, fibroblasts, endothelial cells, sperm cells, and reticular testicular epithelial cells. Studies have revealed that infection with SARS-CoV-2 generates viral orchitis, which is manifested as a large number of degraded germ cells and Sertoli cells, cellular swelling, vacuolization, increased apoptosis, and infiltration of inflammatory cells; the latter includes T lymphocytes, B lymphocytes, and macrophages in the testicular interstitium and seminiferous tubules^10,12^. Jorge Hallak^42^ and colleagues observed distinctive changes, including thickening of the basal membrane of seminiferous tubules and vascular alterations. Regions with thickened basal membranes exhibited fibroblasts simultaneously expressing SARS-CoV-2 N protein-positive immune markers and viral particles, indicating that infected fibroblasts might trigger extracellular matrix deposition in the seminiferous tubules. Furthermore, they provided the first description of vascular changes in testicular tissue of patients with COVID-19. All cases showed congestion and endothelial swelling, with five cases displaying fibrinoid thrombi and one case accompanied by venous thrombosis. Since viral particles and antigens were scarcely detected in endothelial cells, the authors suggested that virus-induced endothelial changes may not be the primary mechanism for thrombotic alterations. Testicular vascular changes were predominantly attributed to systemic alterations associated with COVID-19, such as refractory hypoxemia, multisystem thrombosis, and secondary infections. Ischemia of the testicles due to shock and thrombosis may lead to detachment of seminiferous tubule cells from the basal membrane, increased apoptosis, consequently impairing spermatogenesis. Considering that most investigators have not detected SARS-CoV-2 RNA in semen, we hypothesize that the reduction in sperm quality after infection may not be attributable to the virus’s direct effect on sperm, but that it is the orchitis and injury to the seminiferous epithelium caused by SARS-CoV-2’s actions on the testes that may be responsible for the reduction in semen parameters in male patients. In our study, 91.4% of the patients showed symptoms that were classified as mild, with 8.6% of patients manifesting moderate symptoms, and 93.1% of the men only felt mild symptoms. And none of the patients reported any testicular discomfort. These results cause us to believe that orchitis may not have been present in the patients enrolled in our study, and this will be confirmed in the future by testicular puncture and biopsy. Interestingly, consistent with Jorge Hallak^43^ et al.’s study, none of the 26 mild to moderate COVID-19 patients they investigated complained of testicular discomfort, and testicular ultrasound did not reveal the presence of orchitis. This suggests that orchitis may be present in more severe COVID-19 cases and is often accompanied by testicular pain or discomfort. Surprisingly, ultrasound indicated signs of epididymitis in 42.3% of males. Up to 80% of epididymitis cases are caused by bacterial infection^44^, while viral epididymitis is often challenging to detect and prone to misdiagnosis. In symptomatic patients, ultrasound patterns of viral epididymitis resemble those of bacterial epididymitis, but the former typically presents clinically with orchitis, where the testis is usually the first affected organ, followed by epididymal inflammation or abnormalities^45^. Isolated viral epididymitis is relatively uncommon. In this study, the discovery of radiological epididymitis reveals the potential for asymptomatic damage that may be overlooked in the clinical assessment of SARS-CoV-2-infected males. Even with a thorough physical examination by experienced surgeons, ultrasound assessment is irreplaceable for detecting potential subclinical epididymitis, and epididymal injury may have adverse effects on sperm parameters. Therefore, clinicians should be attentive to this condition.

In addition to testicular and epididymal inflammation, researchers found reduced testosterone levels in reproductive-age males with SARS-CoV-2 infection^42,46,47^. This aligns with the pathological features of interstitial cell damage found in orchitis, as testosterone is primarily produced in interstitial cells and regulates spermatogenesis. Another study on changes in testicular endocrine function in 119 reproductive-age males with SARS-CoV-2 infection indicated elevated LH levels and a decreased testosterone/LH ratio^37,48,49^. This highlights the complexity of male hormone level regulation. Interestingly, in another study by Jorge Hallak^50^ and colleagues, inoculation of the SARS-CoV-2 nucleocapsid protein into the testes, epididymis, prostate, and seminal vesicles of rats did not show significant histological changes. However, the treated group exhibited higher sperm counts and lower testosterone levels, while LH levels did not change significantly. This suggests that simple immunization does not affect male reproductive tract tissues, and the decrease in testosterone levels is not a direct result of viral invasion, possibly involving mechanisms mediated by the testis. Given the systemic and complex regulation of male hormone production, more relevant research may be needed to confirm the pathogenic mechanisms of SARS-CoV-2 antigens in the human testicular microenvironment, especially regarding the functional impact on interstitial cells of the testis. Further studies are required to elucidate the relationship between SARS-CoV-2 nucleocapsid protein, immune response, and regulation of testicular hormone production. Moreover, the decline in sperm parameters was significantly attenuated within 1 month after SARS-CoV-2 infection, indicating that the infection may directly affect spermatids during the developmental process, as normal spermatogenesis requires approximately 72 days^51^.

### Is fever the main factor influencing sperm quality after SARS-CoV-2 infection?

Fever is a risk factor for the diminution in sperm parameters after SARS-CoV-2 infection. As a common symptom of viral infection, fever can temporarily disturb spermatogenesis^52^, and fever is also a common symptom of SARS-CoV-2 infection^53,54^, with more than 80% of patients experiencing fever^55^. As the testes require a temperature below that of core body temperature to maintain normal spermatogenesis, even mild heat stress will lead to germ cell death and impairment of spermatogenesis (albeit for a limited period)^56^. Authors have demonstrated that semen quality was affected by fever-related illnesses, as fever during meiotic or post-meiotic periods reduced sperm concentration by 32.6% and 35%, respectively^52^. One study involving 18 patients with SARS-CoV-2 infection revealed that patients who were febrile during their infection exhibited reduced sperm concentration, quantity, and motility after recovery relative to individuals with a normal body temperature^54^. Another research group also described lowered sperm count and progressive motility in the febrile vs. non-febrile group^15^. In our study, we assigned the 58 patients with SARS-CoV-2 infection to two groups: one with low-grade fever and the other with moderate-to-high fever, but noted no significant differences between the two groups in terms of age, BMI, time of semen examination before and after infection, duration of disease, or other clinical symptoms in addition to fever. The sperm parameters in the low-grade-fever group showed a decreasing tendency after infection that was not statistically significant, while those in the moderate-to-high fever group showed significant drops in sperm concentration; in the proportions of normal sperm, sperm showing normal motility and progressive motility; and in sperm survival rate compared with prior to SARS-CoV-2 infection. The proportions of spermatozoa with head defects and of immotile sperm were also significantly increased. These results indicate that fever may be a risk factor for reduced sperm parameters after SARS-CoV-2 infection. In addition, the mean maximal temperature and fever duration in the group with moderate-to-high fevers were significantly higher than the same indices in the low-grade fever group, and this may be a reason for the greater declines in the sperm parameters in this group. These findings suggest that controlling fever severity and reducing fever duration may be useful for the recovery of sperm parameters in patients with moderate-to-high fevers. As to the reasons for fever-induced decreases in sperm parameters, we hypothesize three distinct factors. First, prolonged high temperatures during the viral infection period can exaggerate inflammatory reactions, increase damage to the blood–testis barrier, and further exacerbate viral invasion and injury to the testes^13^. Second, fever and systemic inflammation caused by SARS-CoV-2 infection may affect luteinizing hormone and testosterone secretion, leading to changes in semen parameters^57^. Third, fever can also induce oxidative stress, thus increasing oxidative damage to sperm in the seminiferous epithelium. Therefore, in the clinical diagnosis and treatment of COVID-19 patients, attention should be given to the control of fever.

### Energy metabolism and recovery after infection

In addition to the negative impacts of inflammation and fever on sperm parameters, our unpublished data suggest that ACE2 plays a critical role in the energy metabolism of sperm. After the deletion of ACE2, for example, we noted significantly reduced mitochondrial function and ATP levels in mouse sperm. Therefore, changes in ACE2 expression after SARS-CoV-2 infection may constitute a reason for the reduction in sperm motility, but this hypothesis requires further confirmation. From the perspective of time, our study indicated that within 30 days after infection with SARS-CoV-2, sperm parameters (including total sperm count, sperm concentration, total motility, and progressive motility) were significantly reduced, followed by recovery within 30–60 days. After 60 days, there were no differences in sperm motility, viability, or the proportion of normal sperm compared with levels before SARS-CoV-2 infection. However, the recoveries of sperm count and concentration remained slightly slower, with no statistical difference observed after 90 days. Considering that the complete spermatogenic cycle in normal humans is 72 days, we posit that the negative impacts of SARS-CoV-2 on sperm viability and motility gradually lessen and that sperm recover after one cycle of spermatogenesis as the damaged sperm will be replaced by developing healthy sperm. Due to the presence of damaged and dead germ cells at different developmental stages caused by inflammation, immune reactions, and heat stress after SARS-CoV-2 infection, we expect the lowered sperm count and concentration to continue until the numbers of spermatogonia are restored by mitosis (which requires a predictably longer period of time). In summary, our results indicate that the negative impacts of SARS-CoV-2 infection on male sperm parameters are reversible and that the impairment of spermatogenesis is gradually repaired during an extended recovery period. All sperm parameters then basically return to normal 90 days after infection, and this is consistent with previously published studies.

### Current research and our new contributions

While there have been numerous studies on the impact of SARS-CoV-2 infection on male reproduction and sperm quality, including cohort studies, prospective investigations, retrospective analyses, and meta-analyses, the heterogeneity in research methods, complexity of included populations, limited sample sizes, and variations in the duration of studies have made it challenging to reach consistent conclusions. Therefore, alongside our current study, we provide a comprehensive review of the existing research landscape in this field, aiming to contribute a meaningful piece to the scientific puzzle. Based on the published studies, we tend to conclude that SARS-CoV-2 has a negative impact on sperm parameters in the short term. This is supported not only by the “material basis” of SARS-CoV-2 infecting testicular cells through TMPRSS2 and ACE2^58^, but also by recent meta-analysis results^18,59–61^. Interestingly, there are discrepancies between case–control and self-controlled studies. Case–control studies show significant debates on the impact of SARS-CoV-2 infection on semen volume, sperm concentration, and sperm vitality, with half of the studies suggesting an impact and the other half indicating no effect. However, more studies suggest a decrease in total sperm count, with no significant effect on forward sperm movement^18,59–61^. In self-controlled studies, two meta-analyses^59,60^ suggest a significant decrease in various sperm parameters after SARS-CoV-2 infection, aligning more with our current findings. This may be attributed to the self-controlled design eliminating individual differences, resulting in higher study efficiency and consistency with the same sample size. Notably, in the four self-controlled studies included in the first subsection of our results, three showed no significant impact of SARS-CoV-2 infection on sperm parameters. However, this is not contradictory to our earlier conclusion, as the semen collection times in these three studies exceeded 3 months post-recovery, while the only study showing a significant impact had an average semen collection time of 51 days. Consistent with our current study's conclusion, the most substantial decrease in sperm parameters occurred within 30 days after SARS-CoV-2 infection, followed by a gradual recovery after 30 days, with no significant differences 90 days post-infection.

Building on the current research foundation, our study offers several new insights in this field. Firstly, the literature review in the first part of our study summarizes the current research status, combining it with our prospective investigation to provide readers with a panoramic view of the impact of SARS-CoV-2 on sperm parameters and male reproductive health. The self-controlled design of our study eliminates individual differences, achieving higher research efficiency and accuracy with a sample size of 58 cases and multiple samplings at different time points post-recovery. Additionally, the use of linear mixed-effects models for analyzing repeated measurements contributes to improved test efficiency, offering higher-quality research evidence. Secondly, while existing conclusions on the impact of COVID-19 on sperm parameters mostly focus on significant differences in semen volume, sperm concentration, and total sperm count, with seemingly no significant difference in forward sperm motility, our study supplements these findings by examining the influence on forward sperm motility. Furthermore, we compare changes in sperm morphology before and after infection, an aspect less explored in other studies. Thirdly, our study collects semen samples within 1 year before COVID-19 and at various time points (1 month, 2 months, 3 months, and up to 109 days) post-infection. This systematic analysis over the time axis provides a comprehensive understanding of the short-term and long-term effects of SARS-CoV-2 infection on sperm parameters and the recovery process post-infection. Fourthly, we meticulously control for confounding factors, excluding the impact of non-COVID febrile illnesses, sleep deprivation, drug use, smoking, alcohol consumption, steroid hormone medication, testicular diseases, and age on semen quality. This comprehensive approach enhances the reliability of our study results. Additionally, the detailed collection of clinical information from patients, including various symptoms, allows for subgroup analysis based on different symptom categories, especially high, moderate, and low fever groups, offering a more comprehensive exploration of factors influencing semen parameters amid confounding variables. When analyzing the impact of fever on sperm quality, we conduct comparisons across different age groups and BMI, excluding the influence of factors other than fever, thereby enhancing study accuracy—a consideration not explicitly mentioned in the cited studies. Fifthly, the impact of COVID-19 on male fertility remains unclear, with considerable variation in published research results possibly due to small sample sizes and population heterogeneity. Our study provides research evidence from a population in western China, contributing to a more complete understanding of the impact of SARS-CoV-2 on sperm parameters globally. Lastly, our study includes only mild to moderate infection cases, and all patients did not report testicular discomfort. This suggests that SARS-CoV-2 infection may not necessarily affect sperm parameters through severe orchitis. However, whether these patients have orchitis needs confirmation through ultrasound, and the specific mechanisms require further investigation.

## Methods

### Study participants

The patients included in this study were sourced from the Reproductive Medicine Center of Sichuan Provincial Maternity and Child Health Care Hospital. They underwent preconception medical examinations or were males with potential assisted reproductive needs. Among this population, some individuals required assisted reproductive treatment for their partners, potentially undergoing repeated semen analyses before and after enrollment. The patients were diagnosed with SARS-CoV-2 infection through positive nucleic acid or antigen detection. After infection, we notified patients to return for semen collection at different time points (including 1 month, 2 months, and 3 months). Semen was obtained through patient masturbation. We selected patients who had at least one semen analysis before and after infection for inclusion in the study. We extracted the basic clinical information of the patients from the medical records at our hospital. The corresponding self-perceived clinical symptoms were extracted using a pre-designed questionnaire. The patients were 18–65 years of age; we excluded individuals outside this range. Moreover, any other diseases or abnormalities that could reduce infertility were eliminated, including radio-chemotherapy, varicocele, inflammation of the testis and epididymis, mumps, congenital factors, and endocrine abnormalities. Patients diagnosed with other febrile diseases after SARS-CoV-2 infection or treated with antiviral medications such as ribavirin and ritonavir that could affect semen parameters were also excluded. Fifty-eight patients with semen evaluations performed before and after SARS-CoV-2 infection were ultimately included in our study. Routine semen analysis was performed by the Male Reproductive Medicine Laboratory of the Sichuan Provincial Maternity and Child Health Care Hospital. Sperm concentration and motility in the fresh semen samples were analyzed using a phase-contrast microscope (Olympus, BX43).

### Methods for routine semen analysis

#### Chinese expert consensus on routine semen analysis

The semen analysis methods in our laboratory refer to the *Chinese Expert Consensus on Routine Semen Analysis*^62^. This consensus was compiled by the Reproductive Laboratory Subcommittee of the Chinese Association of Sexual Medicine based on references of the 5th edition (WHO5)^63^ and 6th edition (WHO6)^64^ of the “WHO Laboratory Manual for the Examination and Processing of Human Semen” and the ISO 23162:2021 “Basic Examination of Semen—Standardization and Testing Methods”^65^.

#### Semen volume

Semen volume was calculated using the weighing method. The weight of a labeled semen collection cup was measured in advance using an electronic balance, followed by another weighing after semen collection. The difference between the two weights represents the semen volume (assuming a semen density of 1 g/ml, the actual average density of semen is about 1.01 g/ml)^66^.

#### Semen liquefaction

The initial ejaculated semen was usually in a gel-like form and starts to liquefy and becomes thinner within a few minutes. As liquefaction continues, the semen became more and better uniform under 37 °C and in a slow-rotating semen collection cup. The liquefied semen samples were taken out from the 37 °C incubator every 15 min to mix and then observe for liquefaction. Record and note the status of liquefaction either when it occurred within 30 min or not even after 60 min.

#### Semen pH value

The semen pH value was immediately tested after liquefaction, with a drop of semen evenly applied to the pH test paper after the mixture. The color in the soaking area was compared with the colors of the standard strip within 30 s. The matched pH value was then read (the pH value of semen from a fertile men should be more than 7.2).

#### Semen viscosity

Semen was drawn into a disposable plastic pipette with a wide diameter (1.5 mm). The pipette was gently squeezed to allow the semen to drop by gravity, and the length of the thread was observed to evaluate semen viscosity. Normal semen forms discontinuous small drops, while the thread might exceed 2 cm in cases of abnormal, which was taken as the criterium to record semen viscosity.

#### Sperm morphology analysis

Sperm staining and morphology analysis were performed according to the 5th edition (WHO5) and 6th edition (WHO6) of the “WHO Laboratory Manual for the Examination and Processing of Human Semen.” Briefly speaking, after complete liquefaction of semen, the semen samples were thoroughly mixed. Using a pipette, 5–10 μl of semen was dropped onto one side of a clean glass slide, immediately contacting the semen drop with the non-sandblasted side of a second glass slide at a 45° angle. The second slide was then slowly dragged along the long axis of the first slide to create a smear. The dried semen smear was slowly immersed in 95% ethanol for fixation for 15 min. Subsequently, the fixed smear was sequentially immersed in the following solutions for different times: 80% ethanol (v/v) for 30 s, 50% ethanol (v/v) for 30 s, distilled water for 30 s, Harris' hematoxylin for 4 min, distilled water for 30 s, acid ethanol immersion 4–8 times (approximately 1 s per immersion), rinsed with running tap water for 5 min, 50% ethanol (v/v) for 30 s, 80% ethanol (v/v) for 30 s, 15 min at least in 95% ethanol (v/v), orange G6 for 1 min, 95% ethanol (v/v) for 30 s, 95% ethanol (v/v) for 30 s, 95% ethanol (v/v) for 30 s, EA-50 green staining for 1 min, 95% ethanol (v/v) for 30 s, 95% ethanol (v/v) for 30 s, 100% ethanol for 15 s, 100% ethanol for 15 s, xylene: ethanol, (1:2) for 1 min, 100% xylene for 1 min. Finally, a drop of sealing gel was added to the glass slide for subsequent sperm morphology analysis. The proportions of normal sperm, sperm with head defects, and sperm with neck and middle piece defects were calculated among 200 sperm. Orange G and hematoxylin dye liquors were obtained from Besso Biotechnology Co., Ltd (BA4035, BA4041, Zhuhai, China).

#### Teratozoospermia index (TZI) and Sperm deformity index (SDI)

Sperm with morphological abnormalities usually have multiple defects (head defects, middle piece or principal piece defects, or a combination of these defects). Detailed examination of the occurrence rate of various morphological abnormalities may be more useful than a single assessment of the percentage of normal morphology sperm, especially in studying the extent of sperm damage in humans^67,68^. By using a multiple-entry system to record each defect in the head, middle piece, and principal piece of the sperm, two indices can be obtained: teratozoospermia index (TZI)^69,70^ and sperm deformity index (SDI)^71,72^. Research has shown that TZI is associated with in vivo fertility^67,70,73^, while SDI is associated with in vitro fertilization^71^. TZI was calculated as the total number of defects divided by the total number of abnormal sperm, with a maximum count of 4 for each abnormal sperm defect, and additional counts of 1 for excess residual cytoplasm in the head, middle piece, and principal piece; SDI was calculated as the total number of defects divided by the total number of sperm (not just abnormal sperm), with the merging of several head defects counted as 1, while middle piece and principal piece defects were counted separately as 1.

#### Detection of sperm agglutination

In the direct immunobead experiment, the microbeads covalently conjugated with rabbit anti-human IgG or IgA immunoglobulins were directly mixed with washed sperm. The binding of microbeads with anti-human IgG or IgA indicated the presence of IgG or IgA antibodies on the surface of the sperm. The experiment was performed according to the standard experimental procedure of the Sperm Agglutination Test Kit for IgG (Boruide Biotechnology Co., Ltd., BRED-012, Shenzhen, China).

#### Sperm survival rate

The sperm survival rate was measured using eosin-nigrosine staining. The experiment was conducted according to the standard experimental procedure of the Sperm Vitality Staining Reagent Kit (Boruide Life Science Technology Co., Ltd., BRED-014, Shenzhen, China). Briefly speaking, 50 μl of semen was mixed with an equal volume of eosin- nigrosine suspension, and a sperm smear was prepared on a glass slide after 30 s. After drying, the stained sperm (dead sperm) and unstained sperm (live sperm) on the slide were counted using a laboratory counter under a microscope, with 200 sperm evaluated for each replicate sample.

#### Sperm concentration and motility analysis

Sperm concentration and motility analysis were performed manually as follows:

1. Sperm motility analysis: After thorough mixing of liquefied semen, 10 μl of semen was immediately placed on a clean glass slide and covered with a 22 mm × 22 mm × 0.4 mm cover glass (resulting in a thickness of approximately 20 μm). After 1 min of standing, a sperm motility assessment was performed, and the dilution factor of semen required for sperm count was determined^63^. Sperm motility was evaluated using an eyepiece with a grid, while the evaluation area was at least 5 mm away from the edge of the cover glass. Sperm with progressive motility were counted first, followed by non-progressive motility in the same grid, and immobile sperm were counted last. At least 5 different views were systematically observed for each sample, and the number of analyzed sperm was more than 200. The difference between the two analysis results within the 95% confidence interval was acceptable, or the sample would be thoroughly mixed again and re-checked.
2. Sperm concentration analysis and calculation of semen dilution factor: Based on the initial estimation of sperm concentration during sperm motility analysis, the semen dilution factor needed for sperm counting was determined^63^. If there was a large difference in the number of sperm in each field of view, it indicated insufficient mixing of the sample, and the sample would be thoroughly mixed again before further analysis. The preparation method of the semen dilution solution was as follows: 50 g of NaHCO3 and 10 ml of 35% formaldehyde solution were added to 1000 ml of distilled water. Optionally, 0.25 g of thymol blue or 5 ml of saturated methylene blue solution (> 4 mg/ml) was added to enhance the background and make the sperm heads clearer. The solution was stored at 4 °C, for at most 1 year using. The sperm concentration was calculated based on the volume of the counting chamber (depth × area) and the dilution factor.

### Statistical analysis

Continuous data were determined to be normally distributed with the Shapiro–Wilk’s test. Normally distributed and non-normally distributed data are presented as mean (standard deviation, SD) or median (interquartile range, IQR), respectively, and analyzed using the Student’s *t* test or Kruskal–Wallis test to compare between/among groups. Categorical data are presented as frequency (percentage, %), and differences in rates were compared using the Chi-squared test. When the expected count was lower than 5, we adopted Fisher’s exact-probability test to compare the differences in rates. The Wilcoxon signed-rank test and McNamar’s test were used in dependent groups to compare semen parameters before and after SARS-CoV-2 infection. Because we repeated semen parameters over time, we assessed the differences in change from before SARS-CoV-2 infection to days ≤ 30, 31–60, 61–90, and > 90 after testing positive for COVID-19 using a linear mixed-effects model. In the linear mixed-effects model, we entered the time of the semen quality test as the fixed effect, and participant ID was entered as the random effect. We completed all analyses using R-4.2.0 (R Foundation for Statistical Computing), and analyses were two-sided, with P values < 0.05 indicating statistical significance.

### Ethics approval and consent to participate

The study was conducted according to the guidelines of the Declaration of Helsinki and approved by the Medical Ethics Committee of Sichuan Provincial Maternity and Child Health Care Hospital (20230626-188), and all patients provided signed informed consent.

### Informed consent

All patients provided signed informed consent for their data for publication and we promise to strictly protect patient privacy.

### Supplementary Information

Supplementary Information.

## Data Availability

The data that support the findings of this study are available from the corresponding author upon reasonable request and all of them were presented in the submitted manuscript.
