# Supplementary material for: Systematic analyses of the factors influencing sperm quality in patients with SARS-CoV-2 infection
Source: Sci Rep. 2024 Apr 7;14:8132. doi: 10.1038/s41598-024-58797-y (PMC10999436; doi:10.1038/s41598-024-58797-y)
Supplement: Supplementary file 1 — Supplementary Information. [file 41598_2024_58797_MOESM1_ESM.docx]

**Supplementary materials**

**
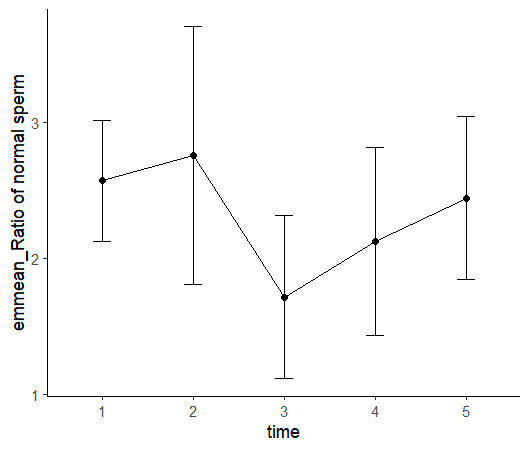
**

**Figure S1. The estimated marginal means(EMMs) of all the observations of normal sperm ratio at different time points of the patients.** 1 represents pre-SARS-CoV-2 infection; 2 represents the observation time was within 30 days after infection; 3 represents the observation time was between 31 and 60 days after infection; 4 represents the observation time was between 61 and 90 days after infection; 5 represents the observation time was more than 91 days.


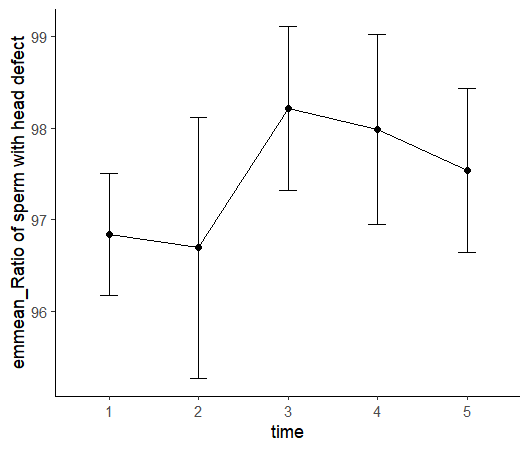


**Figure S2. The average values of all the observations of sperm ratio with head defect at different time points of the patients.** 1 represents pre-SARS-CoV-2 infection; 2 represents the observation time was within 30 days after infection; 3 represents the observation time was between 30 and 60 days after infection; 4 represents the observation time was between 60 and 90 days after infection; 5 represents the observation time was more than 90 days.

**
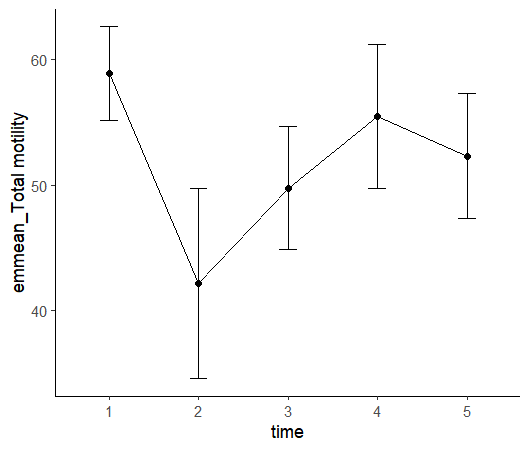
**

**Figure S3. The average values of all the observations of total motility at different time points of the patients.** 1 represents pre-SARS-CoV-2 infection; 2 represents the observation time was within 30 days after infection; 3 represents the observation time was between 30 and 60 days after infection; 4 represents the observation time was between 60 and 90 days after infection; 5 represents the observation time was more than 90 days.

**
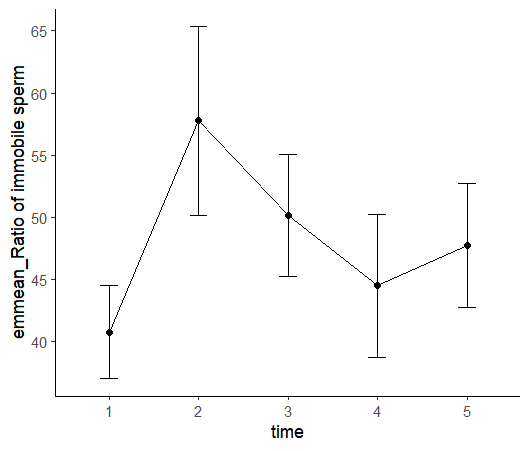
**

**Figure S4. The average values of all the observations of immobile sperm ratio t at different time points of the patients.** 1 represents pre-SARS-CoV-2 infection; 2 represents the observation time was within 30 days after infection; 3 represents the observation time was between 30 and 60 days after infection; 4 represents the observation time was between 60 and 90 days after infection; 5 represents the observation time was more than 90 days.

**
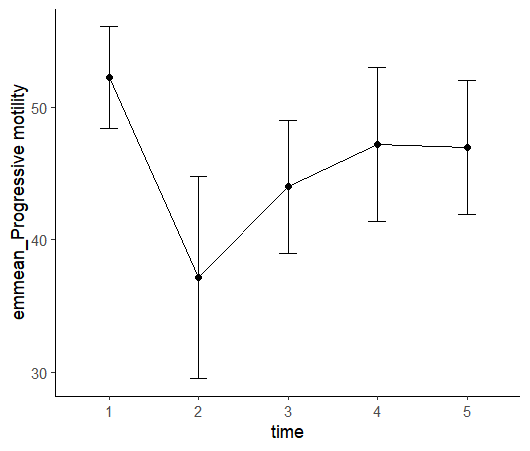
**

**Figure S5. The average values of all the observations of progressive motility at different time points of the patients.** 1 represents pre-SARS-CoV-2 infection; 2 represents the observation time was within 30 days after infection; 3 represents the observation time was between 30 and 60 days after infection; 4 represents the observation time was between 60 and 90 days after infection; 5 represents the observation time was more than 90 days.

**
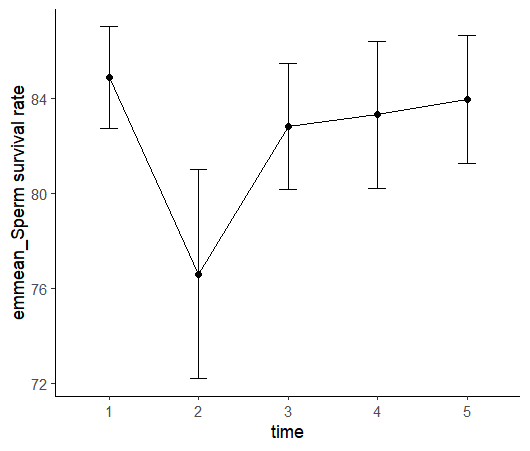
**

**Figure S6. The average values of all the observations of sperm survival rate at different time points of the patients.** 1 represents pre-SARS-CoV-2 infection; 2 represents the observation time was within 30 days after infection; 3 represents the observation time was between 30 and 60 days after infection; 4 represents the observation time was between 60 and 90 days after infection; 5 represents the observation time was more than 90 days.

**
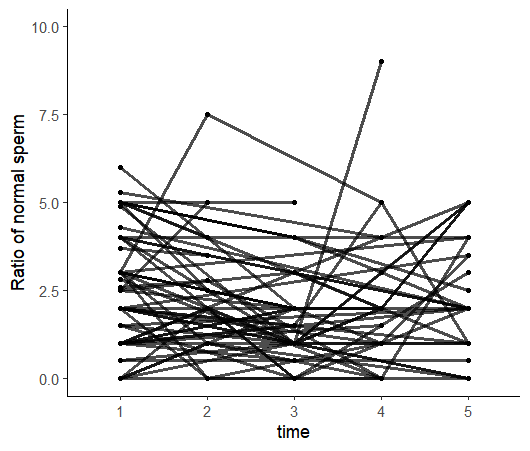
**

**Figure S7. Observations of normal sperm ratio at different time points of the patients.** 1 represents pre-SARS-CoV-2 infection; 2 represents the observation time was within 30 days after infection; 3 represents the observation time was between 30 and 60 days after infection; 4 represents the observation time was between 60 and 90 days after infection; 5 represents the observation time was more than 90 days.

**
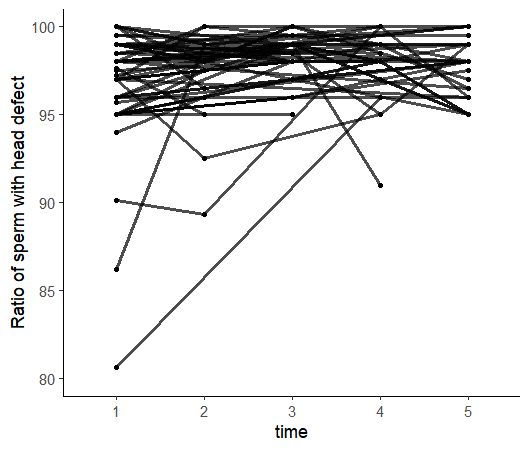
**

**Figure S8. Observations of sperm ratio with head defect at different time points of the patients.** 1 represents pre-SARS-CoV-2 infection; 2 represents the observation time was within 30 days after infection; 3 represents the observation time was between 30 and 60 days after infection; 4 represents the observation time was between 60 and 90 days after infection; 5 represents the observation time was more than 90 days.

**
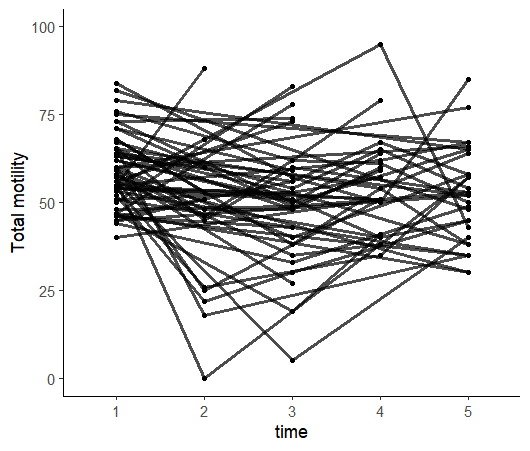
**

**Figure S9. Observations of total motility at different time points of the patients.** 1 represents pre-SARS-CoV-2 infection; 2 represents the observation time was within 30 days after infection; 3 represents the observation time was between 30 and 60 days after infection; 4 represents the observation time was between 60 and 90 days after infection; 5 represents the observation time was more than 90 days.

**
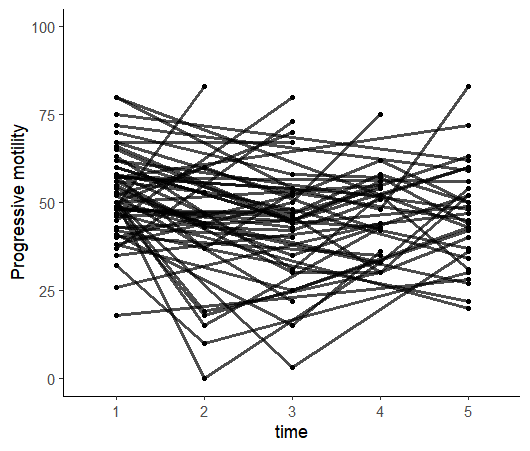
**

**Figure S10. Observations of progressive motility at different time points of the patients.** 1 represents pre-SARS-CoV-2 infection; 2 represents the observation time was within 30 days after infection; 3 represents the observation time was between 30 and 60 days after infection; 4 represents the observation time was between 60 and 90 days after infection; 5 represents the observation time was more than 90 days.

**
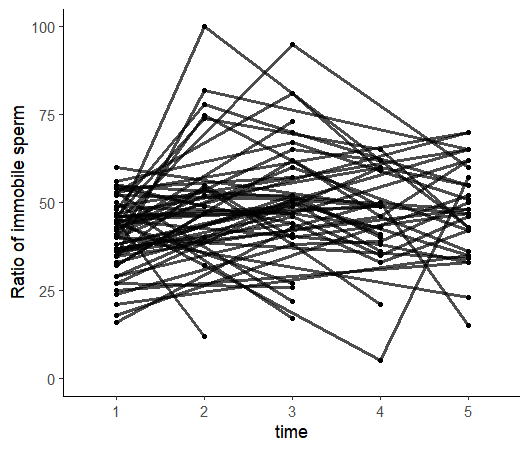
**

**Figure S11. Observations of immobile sperm ratio at different time points of the patients.** 1 represents pre-SARS-CoV-2 infection; 2 represents the observation time was within 30 days after infection; 3 represents the observation time was between 30 and 60 days after infection; 4 represents the observation time was between 60 and 90 days after infection; 5 represents the observation time was more than 90 days.

**
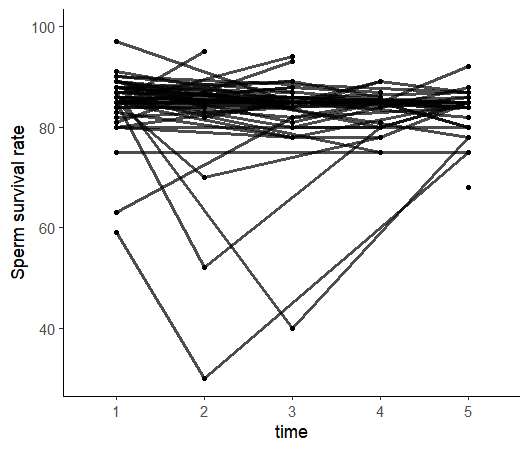
**

**Figure S12. Observations of sperm survival ratio at different time points of the patients.** 1 represents pre-SARS-CoV-2 infection; 2 represents the observation time was within 30 days after infection; 3 represents the observation time was between 30 and 60 days after infection; 4 represents the observation time was between 60 and 90 days after infection; 5 represents the observation time was more than 90 days.


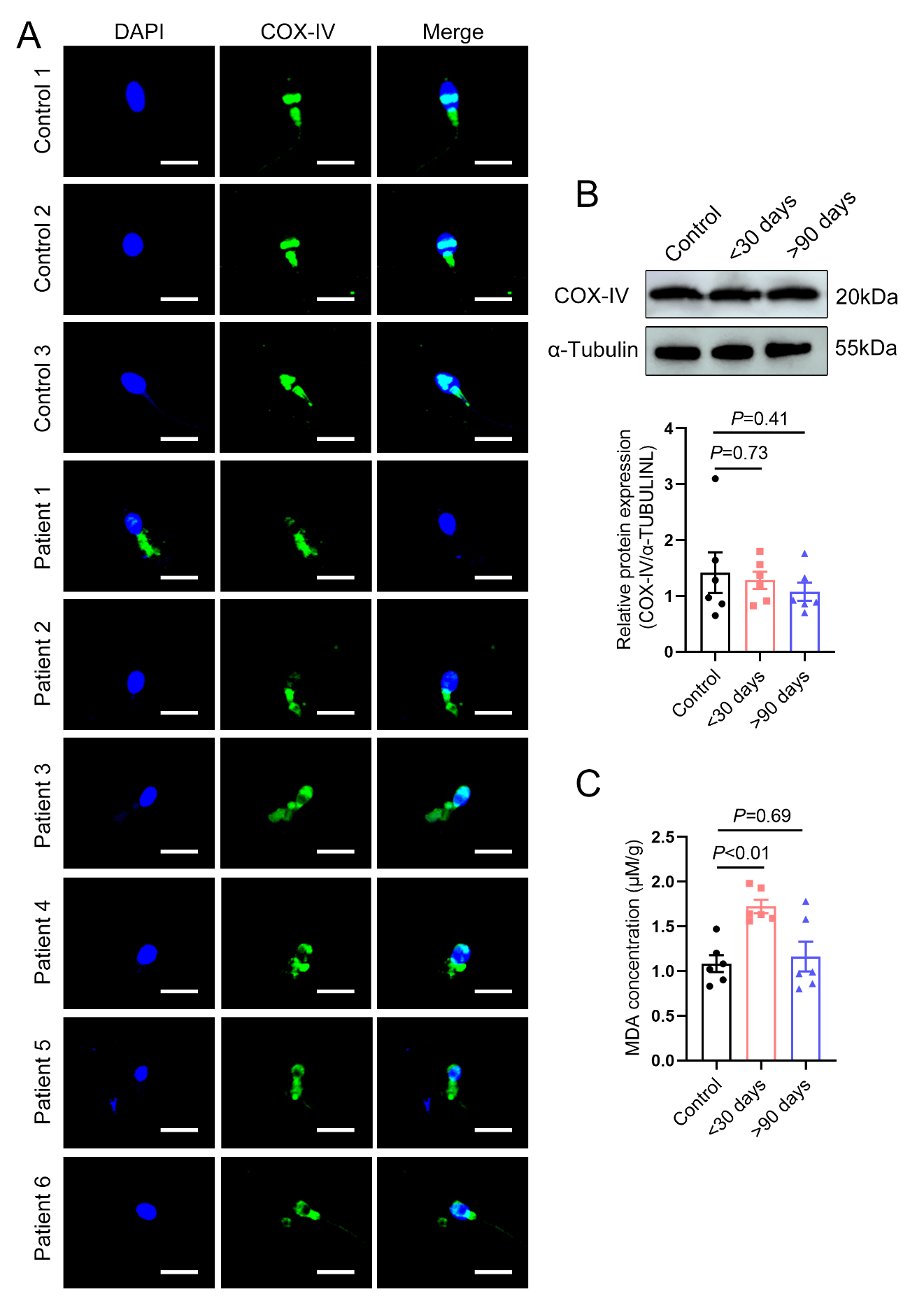


**Figure S13.** (A) The distribution of COX-IV in SARS-CoV-2 infected patients' sperm was more diffuse compared to the control group, Patient 1-3, within 30 days post infection; Patient 4-6, more than 90 days post infection. Scale bars, 5 µm. (B) The expression of COX-IV showed no significant difference from the control group either within 30 days or 90 days post infection. Student’s t test, n=6, error bars, SEM. (C) The MDA concentration in patients' sperm significantly increased within 30 days post infection, but showed no significant difference after 90 days compared to the control group. Student’s t test, n=6, error bars, SEM.

**Table S1. Comparison of basic clinical characteristics between patients with low fever and those with moderate to high fever**

| Variable | Low fever  (n=19) | Moderate or high fever (n=39) | *P* value |
| --- | --- | --- | --- |
| Age^1^ | 31.9 (2.98) | 31.5 (3.39) | 0.643 |
| BMI^1^ | 24.2 (3.72) | 23.5 (3.00) | 0.483 |
| Days of semen analysis before SARS-CoV-2 infection^2^ | 72.0 [27.5, 336] | 77.0 [25.0, 206] | 0.492 |
| Days of semen analysis after SARS-CoV-2 infection^2^ | 43.0 [29.0, 56.5] | 51.0 [38.0, 90.5] | 0.101 |
| ≦30 days^3^ | 6 (31.6%) | 7 (17.9%) | 0.256 |
| 30-60days^3^ | 10 (52.6%) | 18 (46.2%) |  |
| >60 days^3^ | 3 (15.8%) | 14 (35.9%) |  |
| Duration of infection^2^ | 4.00 [3.00, 5.50] | 5.00 [4.00, 6.00] | 0.101 |
| Maximum fever temperature^2, 4^ | 37.8 [37.5, 38.0] | 38.6 [38.5, 39.0] | <0.001** |
| Fever days^2, 4^ | 2.00 [1.50, 2.00] | 3.00 [2.00, 3.00] | 0.004** |
| Clinical classification |  |  | 0.161 |
| Mild^3^ | 19 (100%) | 34 (87.2%) |  |
| Moderate^3^ | 0 (0.00%) | 5 (12.8%) |  |
| Self-feeling^4^ |  |  | 0.023* |
| Mild^3^ | 13 (68.4%) | 12 (30.8%) |  |
| Moderate^3^ | 6 (31.6%) | 23 (59.0%) |  |
| Severe^3^ | 0 (0.00%) | 4 (10.3%) |  |
| Muscle soreness^3^ | 17 (89.5%) | 31 (79.5%) | 0.472 |
| Dry cough^3^ | 12 (63.2%) | 24 (61.5%) | >0.999 |
| Sore throat^3^ | 16 (84.2%) | 28 (71.8%) | 0.350 |
| Parageusia^3^ | 8 (42.1%) | 16 (41.0%) | >0.999 |
| Heterosmia^3^ | 4 (21.1%) | 9 (23.1%) | >0.999 |
| Diarrhea^3^ | 3 (15.8%) | 6 (15.4%) | >0.999 |
| Testicular discomfort^3^ | 0 (0%) | 0 (0%) |  |
| Dizziness and headache^3^ | 8 (42.1%) | 20 (51.3%) | 0.914 |
| Expectoration^3^ | 8 (42.1%) | 11 (28.2%) | 0.447 |
| Nasal obstruction^3^ | 6 (31.6%) | 15 (38.5%) | 0.825 |
| Short of breath^3^ | 2 (10.5%) | 12 (30.8%) | 0.113 |
| Nausea and vomiting^3^ | 1 (5.26%) | 5 (12.8%) | 0.653 |
| Eye discomfort^3^ | 0 (0.00%) | 1 (2.56%) | >0.999 |
| Hoarse voice^3^ | 0 (0.00%) | 1 (2.56%) | >0.999 |
| Palpitate^3^ | 1 (5.26%) | 4 (10.3%) | >0.999 |
| Lumbodynia^3^ | 6 (31.6%) | 5 (12.8%) | 0.151 |
| Feeble^3^ | 8 (42.1%) | 18 (46.2%) | 0.992 |
| Chest tightness^3^ | 1 (5.26%) | 5 (12.8%) | 0.653 |

1, represent as mean (standard deviation).

2, represent as median [Quartile 1, Quartile 3]

3, represent as number (Percentage)

4, * represent *P*<0.05, ** represent *P*<0.01

**Supplementary Methods**

**Immunofluorescence staining**

For sperm staining, samples were initially fixed in 4% paraformaldehyde and permeabilized in 0.3% Triton X-100 (Beyotime, P0096-100m, Shanghai, China) for 15 minutes, followed by blocking in 5% bovine serum albumin (BSA) for 30 minutes at room temperature. Subsequently, the slides were incubated overnight at 4°C with primary antibodies against COX-IV (Abclonal, A6564, 1:200), then incubated with secondary antibodies (Abclonal, AS053, 1:100) at room temperature for 60 minutes. Afterwards, the slides were sealed with 4', 6-diamidino-2-phenylindole (DAPI) (Beyotime, P0131, Shanghai, China) and examined under a laser confocal microscope (Olympus, FV1000, Tokyo, Japan).

**Western blot**

The sperm samples were lysed in RIPA lysis buffer (Beyotime, P0013B), supplemented with a protease inhibitor cocktail (Bimake, B14012, Houston, USA), on ice for 60 minutes. Protein concentrations were determined using the BCA method (Thermo, 23227, Waltham, USA), and equal amounts of total protein were separated by electrophoresis using 10% SDS-PAGE (Shanghai Epizyme Biomedical Technology Co., Ltd., PG112, PG113, Shanghai, China). Subsequently, the proteins were transferred onto polyvinylidene difluoride membranes (Millipore, ISEQ00010, IPVH00010, Boston, USA). The membranes were then incubated sequentially with primary antibodies against α-Tubulin (Abclonal, AC007, 1:1000) and COX-IV (Abclonal, A6564, 1:1000) overnight at 4°C, followed by secondary antibodies (Abclonal, AS014, 1:5000) for 60 minutes at room temperature. Finally, the protein bands were visualized using an ECL HRP substrate (Millipore, WBKLS0500, Boston, USA).

**MDA detection**

MDA detection strictly follows the operator manual (Beyotime, S0131S).
